# Phytochemicals Mediate the Expression and Activity of OCTN2 as Activators of the PPARγ/RXRα Pathway

**DOI:** 10.3389/fphar.2016.00189

**Published:** 2016-06-29

**Authors:** Jian Luo, Jian Qu, Rui Yang, Meng-Xue Ge, Yin Mei, Bo-Ting Zhou, Qiang Qu

**Affiliations:** ^1^Department of Pharmacy, Xiangya Hospital, Central South UniversityChangsha, China; ^2^Department of Pharmacy, Second Xiangya Hospital and Institute of Clinical Pharmacy, Central South UniversityChangsha, China; ^3^Institute of Clinical Pharmacology, Xiangya Hospital, Central South UniversityChangsha, China

**Keywords:** phytochemicals, OCTN2, PPARγ, RXRα, activator

## Abstract

Many phytochemicals exert activities as agonists of peroxisome proliferator-activated receptor gamma (PPARγ). This study aims to investigate whether phytochemicals are agonists of the PPARγ/RXRα pathway and modulate the target gene OCTN2. In this study, a luciferase reporter gene system was used to screen novel OCTN2 activators from 39 phytochemicals. Kaempferol, curcumin, and puerarin were found to show the significant PPRE-mediated luciferase activities (>150%) at 20 μM and showed a dose-dependent manner. Phytochemicals also elevated the mRNA and protein expression of OCTN2 in a dose-dependent fashion in colorectal cancer SW480 cells. These induction effects were gradually inhibited by PPARγ antagonist GW9662 in the luciferase reporter gene system and in SW480 cells. Moreover, the results of cell viability assay imply that three phytochemicals probably induce OCTN2 expression leading to the enhanced uptake of its substrate, oxaliplatin, thereby making cells more sensitive to oxaliplatin. The molecular docking study showed the possible binding sites of phytochemicals in PPARγ protein, and all of the docked phytochemicals fitted the same active pocket in PPARγ as troglitazone. All three phytochemicals exhibited hydrogen bonds between their polar moieties and the amino acid residues. Thus, we identified three phytochemicals as PPARγ ligands, which potentiated the expression and activity of OCTN2.

## Introduction

Phytochemicals are derived from plants and play an essential role in long-term health and offer chemical scaffolds for discovering novel drugs against a wide range of therapeutic targets (Ernst, 2003). Many phytochemicals govern the expression of drug-metabolizing enzymes and drug transporters through activating several nuclear receptors (NRs) and binding to the responsive elements within those genes.

The peroxisome proliferator-activated receptor (PPAR) family consists of three subtypes, namely PPAR alpha, beta and gamma, which are encoded by three distinct genes. Ligand-activated PPARγ acts as a transcription factor and assembles into a heterodimer complex with retinoid X receptor alpha (RXRα) or retinoic acid receptor alpha (RARα), binding to the peroxisome proliferator responsive element (PPRE) within the promoter of target genes. Trans-activation could regulate adipocyte proliferation and differentiation (CCAAT/enhancer binding protein alpha, signal transducer and activator of transcription 1), lipid metabolism (GABA receptor modulator, lipoprotein lipase), glucose homeostasis (glucose transporter type 4, c-Cbl-associated protein, insulin receptor substrates 1 and 2) as well as insulin sensitization (tumor necrosis factor-α, leptin, adiponectin; Ricote et al., 1998; Evans et al., 2004; Zoete et al., 2007). Two groups of PPARγ agonists have been reported: one is the synthetic agonists including thiazolidinediones (TZDs; Quinn et al., 2008); the other is the natural agonists such as endogenous ligands and naturally occurring compounds (Belvisi and Hele, 2008). The development of novel and effective PPARγ agonists from naturally occurring compounds with an improved safety profile and fewer side effects is of great interest.

Organic anion/cation transporter 2 (OCTN2) is a member of the solute carrier transporters, which are expressed in human tissues including the kidney, heart, brain, skeletal muscle, small and large intestine, etc. (Tamai et al., 1998; Wu et al., 1998; Peltekova et al., 2004). It has been shown to transport various endogenous substrates (carnitine and choline), as well as various xenobiotics (cimetidine, mildronate, verapamil, pyrilamine, oxaliplatin, imatinib, and cephaloridine; Grigat et al., 2009; Kano et al., 2009; Kato et al., 2009; Jong et al., 2011; Angelini et al., 2013). The occurrence of mutations in *OCTN2* resulted in systemic carnitine deficiency and an early lethality attributed to metabolic abnormalities in the cardiovascular system and skeletal muscle (Scaglia et al., 1998). It was reported that the heterodimer of PPARγ/RXRα could trigger human OCTN2 expression by binding to the PPRE in the first intron, especially under the effect of troglitazone, which is a conventional ligand of PPARγ (D’Argenio et al., 2010). We speculate that the activation of PPARγ by a natural agonist can trigger the expression of *OCTN2*, which is the PPARγ target gene. In our previous study, luteolin, an agonist of PPARγ, could transactivate *OCTN2*, thereby increasing the transport of oxaliplatin (Qu et al., 2014).

In this study, based on the mechanism of PPARγ-activating OCTN2 expression, we constructed a luciferase reporter gene system for screening novel OCTN2 activators from phytochemicals. The novel PPARγ agonists from herbal compounds supply the basically pharmacological structure which is beneficial for developing more effective ligands. These phytochemicals are also helpful for those patients with OCTN2 deficiency disease or for facilitating the uptake of OCTN2 substrates.

## Materials and Methods

### Chemicals and Reagents

GW9662, troglitazone, oxaliplatin and 39 phytochemicals were purchased from Sigma-Aldrich (St. Louis, MO, USA) and the National Institutes for Food and Drug Control (Beijing, China). TRIzol reagent was obtained from Takara (Dalian, China). Lipofectamine 2000 was obtained from Invitrogen (Carlsbad, CA, USA). All of the primary antibodies were purchased from Abcam Labs (Cambridge, MA, USA).

### Molecular Clones

The expression plasmids of pcDNA3.1-PPARγ and RXRα were constructed. The core sequence of the PPARγ response element (PPRE, AGGTGAAAGGGCA) identified in the first intron of OCTN2 was utilized to construct an oligonucleotide (D’Argenio et al., 2010). A pair of complementary oligonucleotides containing OCTN2 PPRE and flanking sites for *Kpn* I and *Hind* III were annealed and subcloned into the pGL4.23 (luc2) vector digested by *Kpn* I and *Hind* III (Promega, Madison, WI, USA), which contains the minimal promoter (minP) followed by the luciferase reporter gene *luc2* (Promega, Madison, WI, USA; D’Argenio et al., 2010).

### Cell Culture

COS-7 and SW480 cell lines were obtained from the American Type Culture Collection (ATCC, USA). Cells were grown in DMEM containing L-glutamine supplemented with penicillin (100 U/ml), streptomycin (100 g/ml) and 10% (v/v) fetal bovine serum (FBS) at 37°C in a humidified atmosphere with 5% CO_2_.

### Transfection and Dual-luciferase Reporter Assay

COS-7 cells were cultured in a 24-well plate at a density of 1 × 10^5^/well at 37°C. After 8 h, COS-7 cells were co-transfected with pcDNA3.1-PPARγ and RXRα (0.2 μg/well), pGL4.23-PPRE/OCTN2 (0.2 μg/well) or pGL4.23 (0.2 μg/well), as well as pRL-TK (0.02 μg/well) using Lipofectamine 2000 in accordance with the manufacturer’s instructions. In an initial screening assay, cells were washed by PBS and exposed to phytochemicals at 20 μM for 48 h. DMEM medium containing phytochemicals or DMSO was renewed every 24 h. In the dose-dependent assay, transfected cells were exposed to either phytochemicals or troglitazone at various concentrations (1, 2.5, 5, 10, 20, or 40 μM) for 48 h. After being washed twice by PBS, treated cells were harvested and lysed. Then, 20 μl of supernatant was aspirated and used for dual-luciferase activity detection. The luciferase activity is represented as relative light units (RLUs).

### Cell Treatment, RNA Extraction, and Quantitative RT-PCR

SW480 cells were seeded in a 6-well plate at a density of 5 × 10^5^ cells/well and exposed to potent activators from phytochemicals at various concentrations (0, 5, 10, 20, or 40 μM) for 48 h at 37°C. Only DMSO (v/v 0.1%) was added to control cells. DMEM medium containing phytochemicals or DMSO was renewed every 24 h. In the antagonist assay, 20 μM phytochemicals plus a range of concentrations of GW9662 (5, 10, or 20 μM) was incubated with SW480 cells for 48 h. Cells were washed twice by PBS, and total mRNA was extracted by TRIzol reagent (Takara). Two micrograms total RNA was reverse transcribed to cDNA following the manufacturer’s instructions of the first strand cDNA synthesis kit (Takara). The level of OCTN2 mRNA was determined by quantitative RT-PCR using a SYBR Green Kit (Bio-Rad, Hercules, CA, USA). The primers were the following: OCTN2, F: 5′-CCATTGTGACCGAGTGGAACC-3′, R: 5′-ACATTCTTCCGGCCAAACCTG-3′ and β-actin, F: 5′-TTGCCGACAGGATGCAGAAGGA-3′, R: 5′-AGGTGGACAGCGAGGCCAGGAT-3′. The PCR conditions included the following steps: initial denaturation at 95°C for 2 min, followed by 40 cycles at 95°C for 15 s, 60°C for 15 s, and 72°C for 30 s. The relative mRNA levels of OCTN2 were normalized to β-actin and quantitated by the 2^–ΔΔCt^ method (Livak and Schmittgen, 2001).

### Western Blotting

After treatment with three phytochemicals, SW480 cells were lysed, and total proteins were extracted. Concentrations of proteins were determined by the BCA assay (Thermo Scientific, Rockford, IL, USA). Western blotting and the calculation of the relative protein level of OCTN2 were conducted as described in our previous study (Qu et al., 2014).

### Cell Viability

SW480 cells were grown in 6-well plates and treated with either 20 μM phytochemicals or DMSO (v/v 0.1%) for 48 h. DMEM medium containing phytochemicals or DMSO was renewed every 24 h. Cells were washed with PBS and seeded in 96-well plates (2 × 10^4^/well) and then exposed to various concentrations of oxaliplatin for 48 h at 37°C. Next, 20 μl of MTS (tetrazolium salt, Promega) was added to each well, followed by 2 h of incubation at 37°C. The absorbance was analyzed using a Microplate Reader FLX800 at 490 nm (Biotek). The IC_50_ value was calculated by SPSS version 13.0 (Chicago, IL, USA).

### Autodock Analysis

To investigate the possible binding sites of phytochemicals in PPARγ protein, the molecular docking analysis was conducted by Autodock Tools (ADTs) v1.1 and the Autodock v4.0.5 program, including Autogrid and Autodock software (Copyright_1991e2000, the Scripps Research Institute^1^). The co-crystalized structure of PPARγ (PBD ID: 2PRG) was obtained from the RCSB Protein Data Bank (PBD), and 3D structures of the three phytochemicals were downloaded from the PubChem website. The rigid roots of each phytochemical were defined automatically, and the amide bonds were made non-rotatable by ADT. The active site of PPARγ was considered a rigid molecule, whereas the phytochemicals were treated as being flexible. The internal default parameters in the AutoDock software were used to analyze all of the variables, except for the number of docking runs (10), the medium number of energy evaluations on genetic algorithm eGA (2, 500,000) and the maximum number of generations in GA (5000). Following the completion of the docking search, the final compound pose was located by evaluating AutoDock’s empirical scoring function in which the conformation with the lowest docked energy value was chosen as the best. The docking model and hydrogen bonds were predicted by ADT and Pymol software (DeLano Scientific LLC).

### Statistical Analysis

Values are presented as the mean ± SD. Statistical comparisons of data were performed with one-way analysis of variance (ANOVA) followed by a *post hoc* test using SPSS 13.0 (Chicago, IL, USA). *p*-values less than 0.05 (*p* < 0.05) were considered statistically significant.

## Results

### Construction of the PPRE/OCTN2-Luciferase Reporter Gene Assay

**FIGURE 1:**
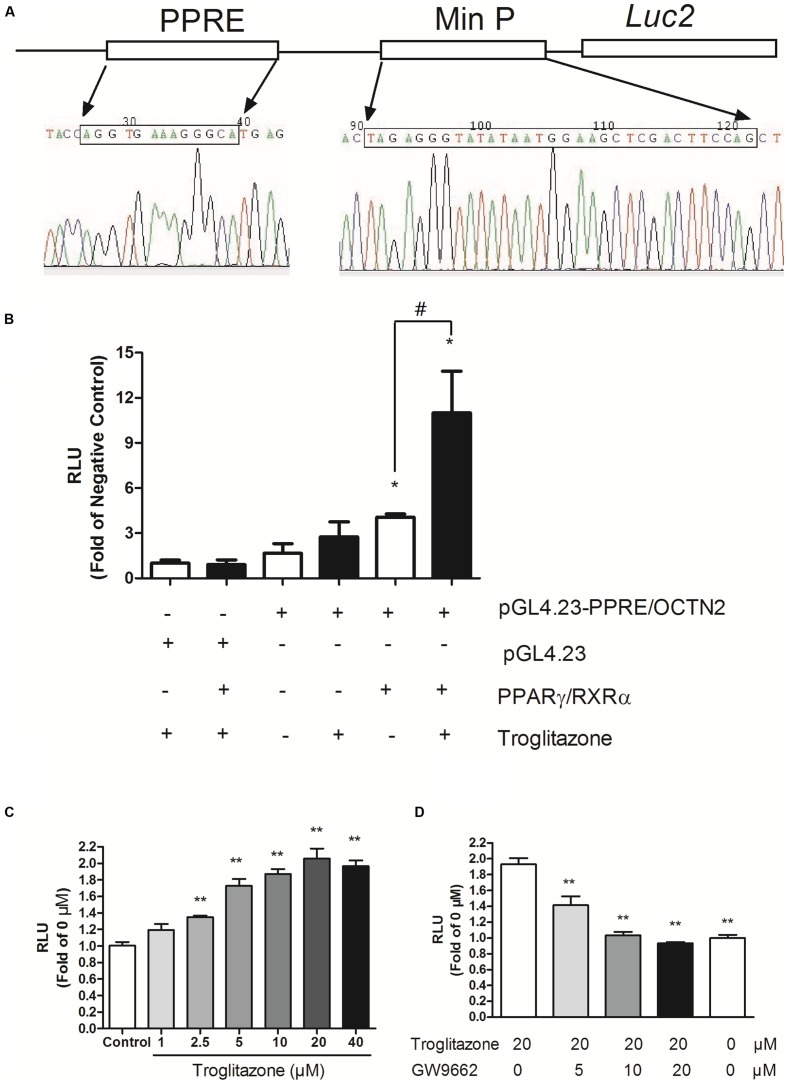
**Construction of PPRE/OCTN2-luciferase reporter gene assay. (A)** The structure of the pGL4.23-OCTN2/PPRE plasmid containing the PPRE of OCTN2, mini promoter and *luc2* gene. **(B)** Identification of Luciferase reporter gene assay. + indicates the transfection of plasmid or the addition of drugs. The relative luciferase units (RLUs) were determined as described in Section “Materials and Methods” indicates untransfected or without addition of drug. *p< 0.05 vs. Group 1; ^#^*p*< 0.05 vs. Group 5. **(C)** Troglitazone (Trog) increased PPRE-luciferase activities in a dose-dependent manner. Data are relative values to the DMSO-treated group. *p< 0.05, **p< 0.01 vs. the DMSO-treated group. **(D)** GW9662 inhibited PPRE-luciferase activities induced by troglitazone. *p< 0.05, **p< 0.01 vs. the 20 μM troglitazone alone without GW9662 group. All values are mean ± SD of three separate experiments.

We successfully constructed the pGL4.23-OCTN2/PPRE plasmid containing the PPRE of OCTN2, min Promoter and *luc2* gene (**Figure 1A**). We transiently transfected the constructs or pGL4.23 into COS-7 cells with co-transfection of either pcDNA3.1-PPARγ/RXRα or empty vector (pcDNA3.1). In cells transiently transfected with pGL4.23-OCTN2/PPRE and PPARγ/RXRα, the luciferase activity increased 3.9-fold (*p* < 0.05) and 11-fold (*p* < 0.05), respectively, which was stimulated by troglitazone compared to cells transfected with pGL4.23 without transfection of PPARγ/RXRα (**Figure 1B**). To detect whether endogenous PPARγ interferes with luciferase activity, only pGL4.23-OCTN2/PPRE was transfected in COS-7 cells with or without exposure to troglitazone. However, troglitazone did not significantly increase luciferase activity through activated endogenous PPARγ (**Figure 1B**).

After transfection with pGL4.23-OCTN2/PPRE and pcDNA3.1-PPARγ/RXRα, COS-7 cells were exposed to troglitazone for 48 h. In **Figure 1C**, troglitazone increased PPRE-luciferase activity in a dose-dependent manner, indicating that it modulated OCTN2 expression. Moreover, to test antagonist activity, transfected cells were exposed to increasing concentrations of GW9662 in the presence of 20 μM troglitazone. The luciferase induction by 20 μM troglitazone was abrogated by GW9662 in a concentration-dependent manner (*p* < 0.01; **Figure 1D**).

### Induction Profile of Phytochemicals on PPRE/OCTN2-Luciferase Activity

**FIGURE 2:**
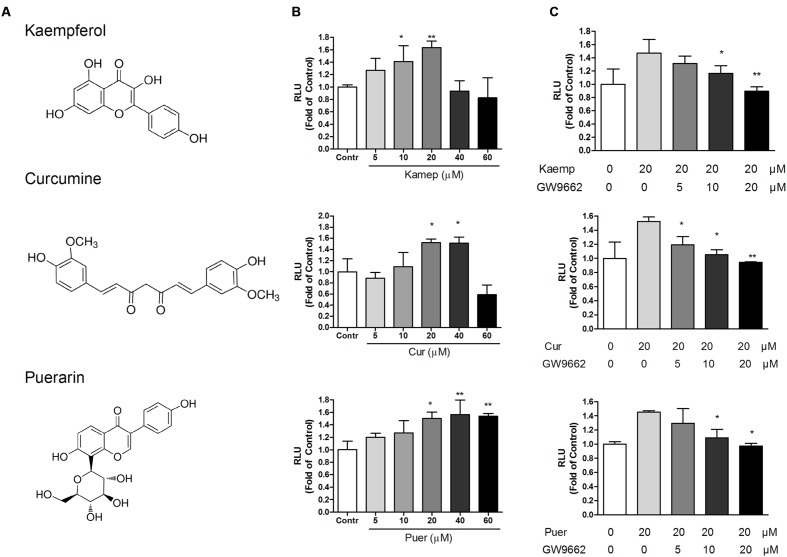
**Effects of phytochemicals and antagonists on PPRE luciferase activities. (A)** The structure of kaempferol, curcumin, and puerarin. **(B)** Effects of three phytochemicals on PPRE/OCTN2-mediated luciferase activities. Control is 0.1% DMSO. *p < 0.05, **p < 0.01 vs. Control group. **(C)** GW9662 antagonized phytochemical-induced luciferase activities. *p < 0.05, **p < 0.01 vs. the 20 μM phytochemicals alone group.

Based on the PPRE/OCTN2-luciferase reporter system, the induction effects of 39 phytochemicals on OCTN2 were investigated at 20 μM. DMSO solution (0.1% v/v) acted as the control (this luciferase activity was considered to be onefold activation), and the results are summarized in **Figure 2** and **Table 1**. Of these compounds evaluated for PPARγ transactivation activity, only three phytochemicals, namely kaempferol, curcumin and puerarin, had a significant inducible effect that was more than a 150% increase compared to the control group. These findings imply that the potent PPARγ activators might activate PPARγ and consequently induce the expression of OCTN2.

**Table 1:** Induction profile of phytochemicals on PPRE/OCTN2-Luciferase activity.

Phytochemical name	Relative RFU fold change (% of blank)	Phytochemical name	Relative RFU change (% of blank)
Troglitazone	188.34 ± 14.85	Ginkgolide A	112.26 ± 12.80
Blank (DMSO)	100.00 ± 8.05	Salidroside	111.98 ± 12.40
Kaempferol	189.89 ± 5.98	Silymarin	109.18 ± 16.55
Curcumin	179.71 ± 13.81	Breviscapine	108.73 ± 19.10
Puerarin	153.04 ± 12.88	Aloe-emodin	105.30 ± 18.72
Salvianolic acid B	143.54 ± 6.01	Echinacoside	104.93 ± 16.62
Isocorynoxeine	138.24 ± 27.39	Ginkgolide C	104.14 ± 9.67
Ursolic acid	133.81 ± 8.23	Ophiopogonin	102.64 ± 12.58
Rutin	131.78 ± 26.51	Protopanaxatriol	98.19 ± 3.54
Daidzin	131.27 ± 44.63	Corynoxeine	97.44 ± 20.92
Polydatin	128.54 ± 57.91	Laburnine	94.35 ± 14.43
Ginsenoside	127.94 ± 29.03	Resveratrol	93.70 ± 4.37
Matrine	122.14 ± 3.05	Quercitrin	81.78 ± 6.36
Hesperidin	119.71 ± 17.87	Ferulic acid	80.27 ± 17.08
Rheinic acid	119.34 ± 30.34	Hyperoside	75.26 ± 4.35
Protopanaxatriol	118.63 ± 1.96	Tanshinone IIA	73.30 ± 22.98
hydroxysaﬄor yellow A	115.91 ± 24.49	Geniposide	71.67 ± 11.39
Ginsenoside Rc	114.15 ± 25.25	Nobiletin	67.54 ± 13.50
Ginkgolide B	113.45 ± 13.75	Osthole	66.99 ± 30.08
Oleanolic acid	113.41 ± 5.65	Berberine hydrochloride	46.28 ± 27.01
Engelitin	112.53 ± 11.77		

### Concentration-Dependent Effects of Phytochemicals on PPRE/OCTN2-Mediated Luciferase Expression

Three phytochemicals at various concentrations were used to determine their dose-dependent effects. In **Figure 2**, we observed that the PPRE-mediated luciferase activity was gradually enhanced in accordance with the increase in phytochemical dose and reached the most induction during exposure of transfected cells to these phytochemicals at 20 and 40 μM (*p* < 0.05). To determine whether the induction effects of potent ligands on OCTN2 were dependent on PPARγ, the antagonist GW9662 was used to co-incubate with phytochemicals and transfected cells. As illustrated in **Figure 2**, phytochemical-induced luciferase activities were gradually inhibited by GW9662 in a concentration-dependent manner. GW9662 (20 μM) completely abrogated the induction effects of the phytochemical.

### Effects of Phytochemicals on mRNA and Protein Levels of OCTN2 in Colon Cancer SW480 Cells

**FIGURE 3:**
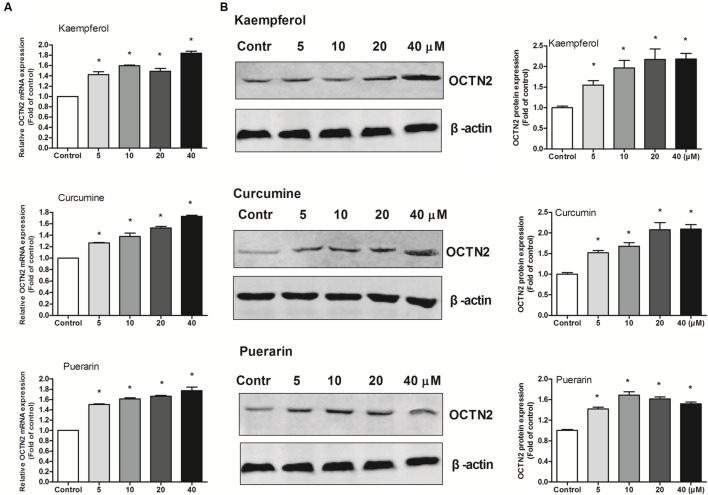
**Effects of phytochemicals on mRNA and protein levels of OCTN2 in colon cancer SW480 cells.** The quantitation and calculation of relative expression of OCTN2 mRNA **(A)** and protein **(B)** were performed as described in Section “Materials and Methods.” The vehicle control was 0.1% DMSO. *p < 0.05 vs. the vehicle control group.

To provide better insight into OCTN2 expression induced by phytochemicals in cells, three phytochemicals were incubated with SW480 cells at various concentrations (5–40 μM). As shown in **Figure 3**, compared to the control group (0.1% DMSO), the three phytochemicals significantly induced OCTN2 mRNA and protein levels in a dose-dependent manner.

### Antagonist GW9662 Blocks the Induction of OCTN2 Expression by Phytochemicals in SW480 Cells

**FIGURE 4:**
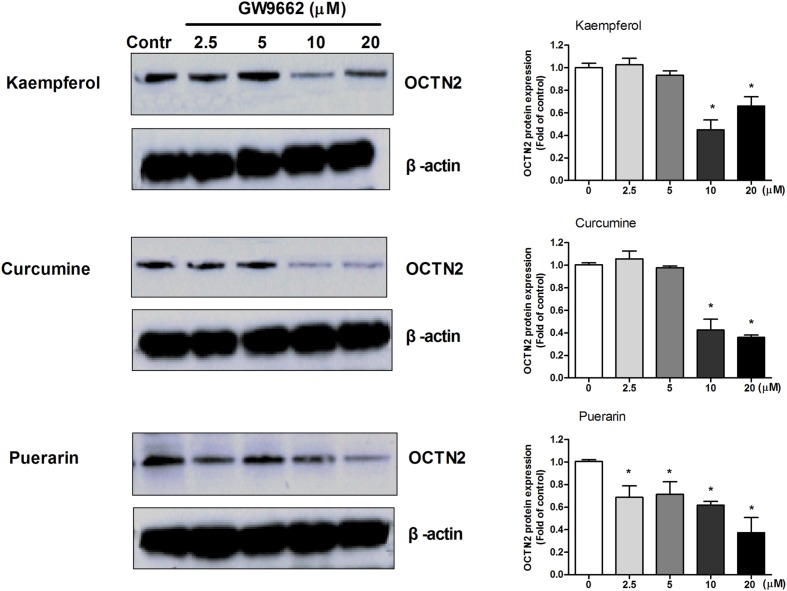
**GW9662 at various concentrations antagonized natural activator-induced protein expression of OCTN2 in SW480 cells.** *p < 0.05, **p < 0.01 vs. the 20 μM phytochemicals alone group.

To determine whether the induction effects of potent ligands on OCTN2 expression were dependent on PPARγ in SW480 cells, a series of concentrations of GW9662 was used to co-incubate with 20 μM phytochemicals and SW480 cells. In **Figure 4**, the relative expression of OCTN2 protein was decreased with the increase in GW9662. The expression of OCTN2 decreased to 0.4- and 0.6-fold of the control group at 10 and 20 μM (*p* < 0.05).

### Impact of OCTN2 Induction by Phytochemicals on Oxaliplatin Cytotoxicity to SW480 Cells

**FIGURE 5:**
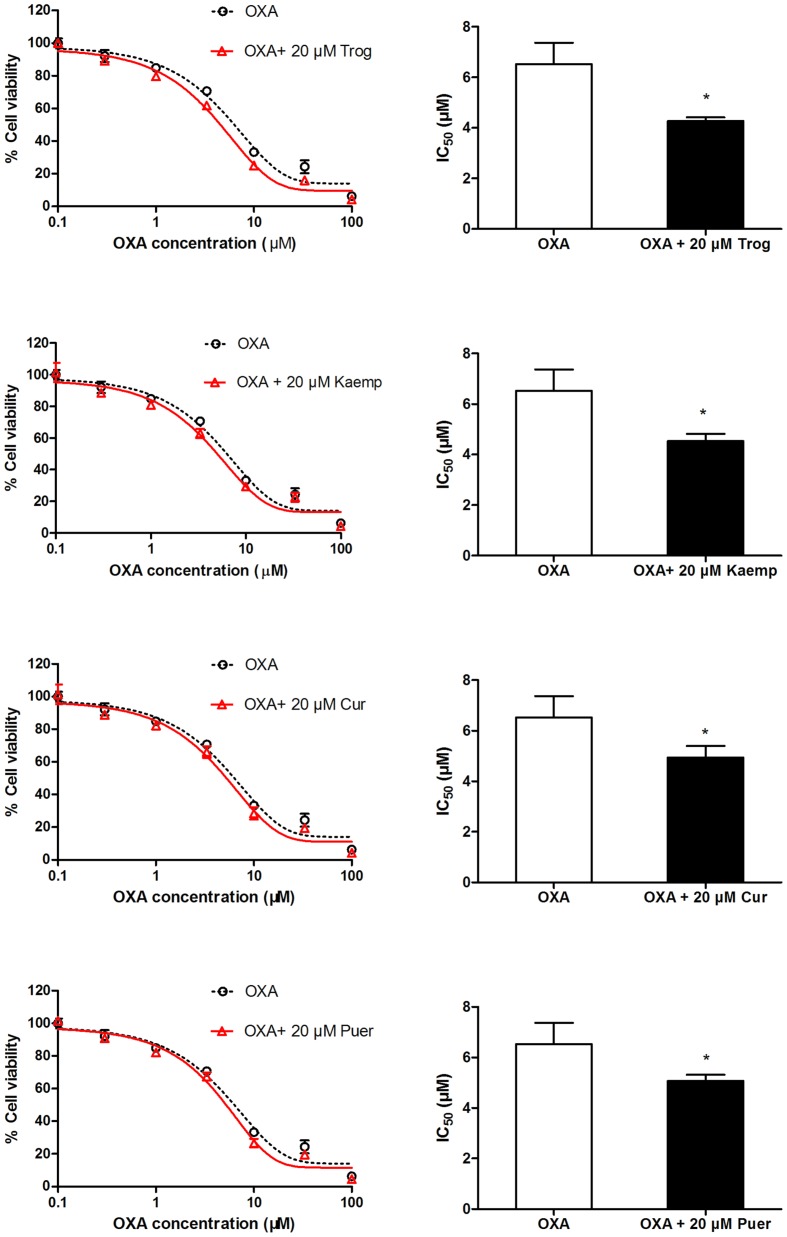
**Influence of the pretreatment of 20 μM phytochemicals on cell viability of SW480 cells against oxaliplatin.** *p < 0.05 vs. the oxaliplatin alone group.

To determine whether phytochemicals make oxaliplatin more cytotoxic to cells by inducing OCTN2 expression and enhancing oxaliplatin uptake, SW480 cells pretreated with the three phytochemicals were exposed to oxaliplatin at various concentrations for 48 h. Cell viability was determined by the MTS assay, and IC_50_ values were calculated. In **Figure 5**, IC_50_ values of cells pretreated by kaempferol, curcumin and puerarin decreased to 0.7-, 0.75-, and 0.77-fold of the group of oxaliplatin alone (*p* < 0.05).

### Molecular Docking Analysis

**FIGURE 6:**
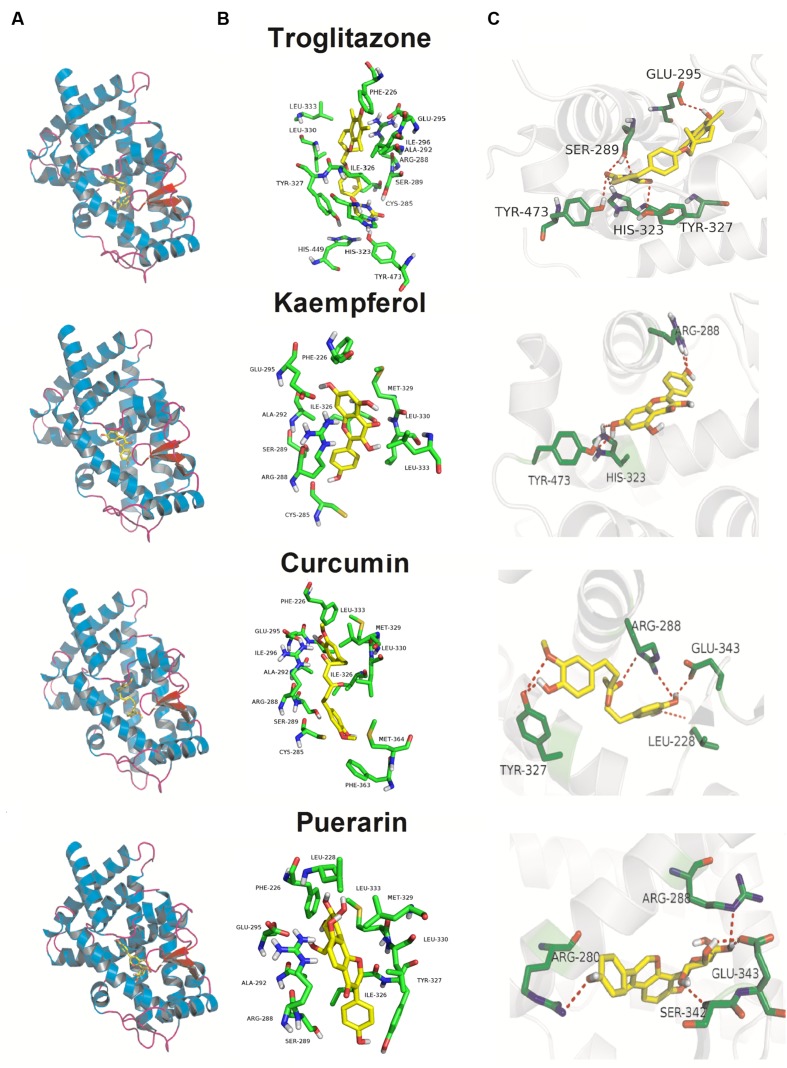
**Docking poses and activity pocket for troglitazone and three phytochemicals in PPARγ protein (PDB ID: 2PRG). (A)** Docking site and **(B)** important residues are labeled and shown as sticks to facilitate the localization of these poses on the active site region in PPARγ. **(C)** The compounds form hydrogen bonds (red dot) with key residues.

As illustrated in **Figure 6**, although, the K_d_ values of docking parameter for kaempferol, curcumin, and puerarin (2.38, 1.21, and 1.29 μM) were lower than that of troglitazone (6.37 nM), all three docked phytochemicals fitted the same active pocket in PPARγ as troglitazone, including amino acids Phe226, Cys285, Arg288, Ser289, Ala292, Glu295, Ile296, His323, Ile326, Tyr327, Met329, Leu330, Leu333, Phe363, Met364, His449, and Tye473. All of them exhibited hydrogen bonds between their polar moieties and the amino acid residues. The 4′-OH substituent of kaempferol formed a hydrogen bond with Arg288, while the 7-OH afforded the second hydrogen bond with the Tyr473 or His323 residue of PPARγ. Curcumin made five potent hydrogen bonds with the chain of the Arg288, Glu343, Leu228, and Tyr327 residues through 3′-OH, 4′-OH, and 4′-OH. Puerarin also made five important hydrophobic interactions with Arg280, Arg288, Glu343, and Ser342 through 4′-OH, 4′′-OH, and 7-OH.

## Discussion

Currently, many studies have considered cell-based transactivation or ligand binding assays to screen for agonists by assessing the transcriptional activity of PPARγ *in vitro*. In our study, we developed a simple screening cell model to identify OCTN2 activators based on a PPRE-luciferase cell model using activation of the PPARγ/RXRα pathway. In the transient transfection, the expression vector of PPARγ/RXRα and the pGL4.23 (luc2) vector, which contains the minimal promoter (minP) and PPRE of OCTN2 followed by the luciferase reporter gene *luc2*, were transfected in COS-7 cells. The cell model is applicable for generating and evaluating new ligands of PPARγ. Their advantages are ease of use, efficiency and reproducibility, which make the luciferase reporter gene assay the most common method for evaluating PPARγ activation. Additionally, this method is beneficial for structure-activity studies and guiding chemical analyses. All phytochemicals are common food and plant sources, and possess different chemical scaffold diversities. In supplementary file 1, we set up the parameters for the screening model as follows: the reading time of 10 s, the seeded cell number of 105 cells/well, the treatment time of 48 h. Based on this model, three phytochemicals have been screened as potent activators of OCTN2. In supplementary file 2, the results of cell viability showed the maximum non-toxic concentration of three natural compounds was 33 μM toward the COS-7 and SW480 cells (>90%). In the subsequent cell experiments, the concentration did not exceed 40 μM.

Mounting evidence suggests that the increase of PPARγ expression and its transcriptional activity as a target play an important role of anti-inflammation and the suppression of inflammation-driven colon cancer (Koeﬄer, 2003; Voutsadakis, 2007). Many naturally occurring elicit their chemopreventive actions against inflammation-induced colorectal cancer through PPARγ activation (Carter et al., 2009). Due to the high expression level of PPARγ, the colon cancer cell SW480 was selected as a tool cell line to further investigate the effect of three phytochemical on the trans-activation of PPARγ and the regulation of OCTN2, as described in our published study (Qu et al., 2014). To detect the transport activity induced by three phytochemicals, oxaliplatin, an excellent substrate of OCTN2, was used in the cell viability assay, which was identified in previous studies (Jong et al., 2011; Qu et al., 2014). Except the cell model, the structure-based docking analysis *in silico* also provide a approach for aiding the performance of the contaction of ligand and PPARγ (Encinar et al., 2015). Although, the K_d_ values of docking parameter for kaempferol, curcumin and puerarin were lower than that of troglitazone, all three docked phytochemicals fitted the same active pocket in PPARγ as troglitazone. These phytochemicals supply the chemical scaffold diversities, and will be new drug leads toward PPARγ. Kaempferol is a flavonoid and is abundant in tea, broccoli, delphinium, witch-hazel, grapefruit, cabbage, kale, beans, endive, leek, tomato, strawberries, grapes, brussel sprouts, apples, and other plant sources (Calderon-Montano et al., 2011; Chen and Chen, 2013). Competitive ligand-binding assays confirmed that kaempferol competed with rosiglitazone at the same binding pocket site as PPARγ (IC_50_ = 23.1, 30 or 49.9 μM; Fang et al., 2008; Zoechling et al., 2011; Jungbauer and Medjakovic, 2012). Kaempferol could significantly improve insulin-stimulated glucose uptake in mature 3T3-L1 adipocytes through PPARγ activation (Lee et al., 2015). Moreover, kaempferol acted as a PPARγ agonist and significantly stimulated PPARγ-induced luciferase activities in a dose-dependent manner in a transient reporter assay (Liang et al., 2001; Fang et al., 2008), which is consistent with our results. To predict the binding mode of kaempferol in PPARγ, the autodock results showed that kaempferol fitted the same active pocket as the positive agonist troglitazone through the formation of hydrogen bonds between polar moieties and amino acid residues. The 4′-OH and 7-OH substituents of kaempferol formed a hydrogen bond with Arg288, Tyr473, or His323 residues of PPARγ. A further antagonist assay showed that GW9662 apparently inhibited kaempferol-induced luciferase activities. In SW480 cells, the PPARγ target gene OCTN2 was elevated by kaempferol at both the mRNA and protein levels in a dose-dependent fashion. These findings imply that kaempferol is a PPARγ agonist and modulates expression of the target gene OCTN2. Cell viability induced by oxaliplatin represents the uptake ability of OCTN2 (Jong et al., 2011). In our study, we also found that pretreatment with kaempferol contributed to higher inhibition of cell growth induced by oxaliplatin, compared to the oxaliplatin alone group. Numerous reports have shown that the consumption of kaempferol, some glycosides of kaempferol and several kaempferol-containing foods may be beneficial in reducing the risk of developing some types of cancer and cardiovascular diseases (Garcia-Closas et al., 1998; Gates et al., 2007; Nothlings et al., 2007; Cui et al., 2008). Evidence suggests that kaempferol is used in combination with some chemotherapeutic drugs and facilitates their cytotoxicity, such as cisplatin (Luo et al., 2010), 5-fluorouracil (Zhang et al., 2008), cytarabine (Nadova et al., 2007), doxorubicin (Sharma et al., 2007), mitoxantrone and the active metabolite of irinotecan (SN-38; Imai et al., 2004). The pharmacokinetic studies of kaempferol in rats found that intravenous administration of 25 mg/kg kaempferol or oral administration of resulted in serum levels of 4.99 and 1.74 μM (Zhang et al., 2015; Qian et al., 2016). Pretreatment of SW480 cells with kaempferol provides new insight into the sensitizing of cancer cells to the cytotoxic effects of oxaliplatin, thereby leading to improved oxaliplatin therapeutic effects or decreased toxicity.

Curcumin, a polyphenol obtained from the turmeric plant, *Curcuma longa*, has received attention as a promising dietary supplement based on its various biological effects such as anti-oxidant, anti-inflammatory, and anti-tumor properties (Srivastava et al., 1995; Maheshwari et al., 2006). Curcumin may act synergistically with the oxaliplatin in gastric cancer by inducing apoptosis via Bcl/Bax-caspases pathways (Zhou et al., 2016). Curcumin also mediates CXC-Chemokine/NF-κB signaling pathway and reverses oxaliplatin-acquired resistance in colorectal cancer cell lines (Ruiz de Porras et al., 2016). In our study, curcumin may only activate PPARγ at high doses (above 20 μM) in a luciferase reporter assay but could elevate the protein and mRNA levels of PPARγ and OCTN2 in a dose-dependent manner in SW480 cells, even at lower doses (5 and 10 μM). However, curcumin has been reported to activate PPARγ (Kuroda et al., 2005; Nishiyama et al., 2005), and it is controversial whether this activation reflects curcumin directly binding to the receptor or is mediated through the effects of receptor expression or levels of the endogenous ligand 15d-PGJ_2_ (Zhang et al., 2006; Narala et al., 2009). More studies are needed to clarify whether curcumin increases PPARγ activity through multiple pathways induced by curcumin. The pharmacokinetic studies of curcumin in humans found that oral administration of 8 g/day curcumin resulted in serum levels of 1.75 μM (Cheng et al., 2001). Pharmacokinetic studies of herb products containing curcumin in long-term use will be helpful to determine the maximum concentration of curcumin in the serum and whether it activates PPARγ and induces OCTN2 expression. Further autodocking results also showed that curcumin fitted the active pocket in PPARγ and made five potent hydrogen bonds with the chain of Arg288, Glu343, Leu228, and Tyr327 residues.

Puerarin is a type of isoflavone and is the main active phytochemical of *kudzu* root, which has strong potential in the treatment of various diseases such as cardiovascular disease, hepatitis, insulin resistance, etc. (Xing et al., 2011; Chen et al., 2012; Guo et al., 2013). Pharmacokinetic studies were conducted in male rats received puerarin (200 mg/kg, po) and the plasma concentrations (C_max_) reached 2.47 μM (Su et al., 2016). Puerarin may act as a chemopreventive and/or chemotherapeutic agent in colon cancer cells HT-29 and hepatocellular carcinoma cell SMMC-7721 by reducing cell viability and inducing apoptosis through bcl-2/caspase3 or mitochondria-dependent pathway (Yu and Li, 2006; Zhang et al., 2014). Although, the direct activation of PPARγ by puerarin has not been found in previous research, many reports found that puerarin promoted PPARγ and target gene expression (Zhao and Zhou, 2012; Guo et al., 2013). Our studies showed that puerarin activated the PPARγ/RXRα pathway and elevated the expression of its target gene OCTN2. Puerarin affected the transport efficiency of OCTN2 toward oxaliplatin through activation of the PPARγ/RXRα pathway and transduction of OCTN2. Puerarin also made five important hydrophobic interactions with Arg280, Arg288, Glu343, and Ser342 through 3′-OH, 4′-OH, and 4′′-OH.

Epidemiological and pre-clinical data suggest that kaempferol, curcumin, and purarin may regulate multiple signaling pathways involved in cancer cell proliferation and apoptosis *in vitro* and *vivo* (Kotecha et al., 2016). In our study, these phytochemicals acted as a sensitizer through the alteration of the pharmacokinetic of oxaliplatin in cancer cells. However, due to the less pharmacokinetic studies in human, whether the plasm concentration of phytochemicals could sufficiently trigger PPARγ/OCTN2 pathway *in vivo* is still not clear. Future pharmacokinetic studies of these phytochemicals *in vivo* will be useful for determining a suitable dose range that will effectively induce OCTN2 expression as well as enhance oxaliplatin effect with less toxicity in other normal tissues.

## Conclusion

We constructed a luciferase reporter gene system for screening novel OCTN2 activators from phytochemicals. Three phytochemicals, namely kaempferol, curcumin and puerarin, were selected, and further biological activity experiments and binding model analyses elucidated that these phytochemicals appeared to mediate OCTN2 expression and activity through PPARγ/RXRα activation.

## Author Contributions

JL, JQ, B-TZ, and QQ designed the study. M-XG and YM did the experiment of cell treatment. JL and QQ did the molecular experiment and autodock study. QQ wrote the paper.

## Conflict of Interest Statement

The authors declare that the research was conducted in the absence of any commercial or financial relationships that could be construed as a potential conflict of interest.

## Footnotes

1 http://www.scripps.edu/mb/olson/doc/autodock
