# Supplementary material for: Phytochemicals Mediate the Expression and Activity of OCTN2 as Activators of the PPARγ/RXRα Pathway
Source: Front Pharmacol. 2016 Jun 29;7:189. doi: 10.3389/fphar.2016.00189 (PMC4925669; doi:10.3389/fphar.2016.00189)
Supplement: Supplementary file 1 [file Data_Sheet_1.DOCX]

**Supplementary file 1**

1. **Optimization of detecting parameters of the luciferase reporter gene model**

Due to the detection time, the seeded cell number, incubation time of drugs and other parameters affect the model, we optimized all parameters. After the reading time of 10 s, the value of relative luciferase activity did not significantly increase and was stable at a plateau, thus, we chose reading time of 10s. Due to too much or too little seeded cell number affect luciferase activity, the maxium activity was found when 10^5^ cells was seeded per well. Regarding to the treatment time, the maxium activity was found after the treatment of troglitazone for 48 hours and 72h. Therefore, we set up the parameters for the screening model of OCTN2 ativators as follows: the reading time of 10 s, seeded cell number of 10^5^ cells/ well, the treatment time of 48 h (Figure S1).


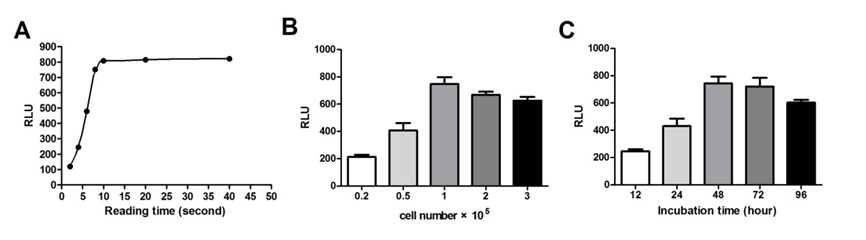


Fig.S1 Optimization of detecting parameters of induced model. The optimization of

(A)，Reading time；(B) cell number；(C)，treatment time。RLU，relative luciferase value.
