# Supplementary material for: Phytochemicals Mediate the Expression and Activity of OCTN2 as Activators of the PPARγ/RXRα Pathway
Source: Front Pharmacol. 2016 Jun 29;7:189. doi: 10.3389/fphar.2016.00189 (PMC4925669; doi:10.3389/fphar.2016.00189)
Supplement: Supplementary file 2 [file Data_Sheet_2.DOCX]

**Supplementary file 2**

After COS-7 and SW480 cell lines were incubated with various concentrations of natural compounds for 48 h, MTS cell viability test were used to identify the growth inhibition of natural compounds on SW480 cells. The maximum nontoxic concentration of the three natural compounds was 33 μM towards the COS-7 and SW480 cells (cell viability > 90%). In the subsequent cell experiments, the concentration did not exceed 40 μM.


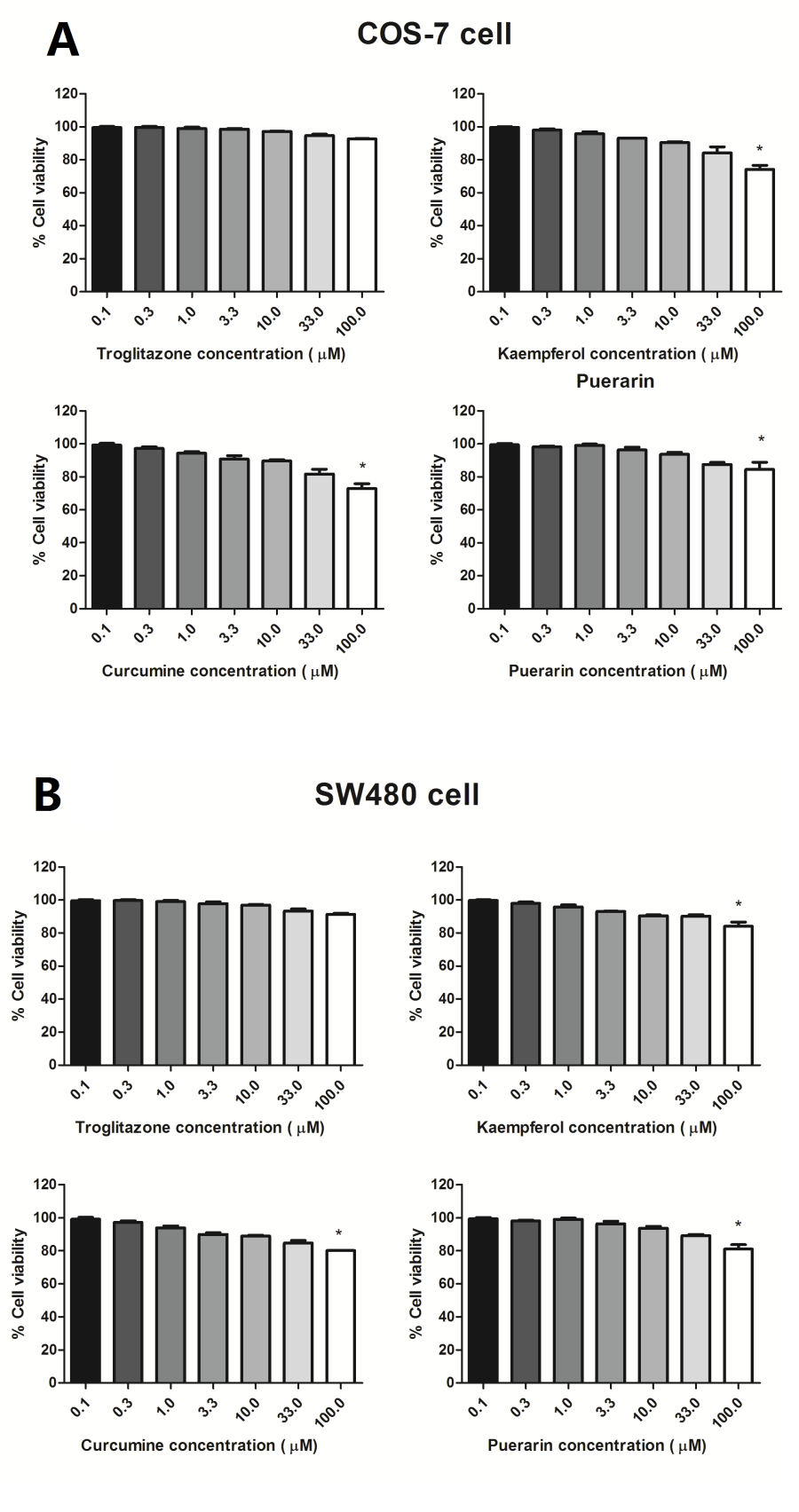


Fig.S2 Influence of natural compounds on cell viability of SW480 cells.
